# Reproducibility of neuroimaging analyses across operating systems

**DOI:** 10.3389/fninf.2015.00012

**Published:** 2015-04-24

**Authors:** Tristan Glatard, Lindsay B. Lewis, Rafael Ferreira da Silva, Reza Adalat, Natacha Beck, Claude Lepage, Pierre Rioux, Marc-Etienne Rousseau, Tarek Sherif, Ewa Deelman, Najmeh Khalili-Mahani, Alan C. Evans

**Affiliations:** ^1^McConnell Brain Imaging Centre, Montreal Neurological Institute, McGill UniversityMontreal, QC, Canada; ^2^Centre National de la Recherche Scientifique, University of Lyon, INSERM, CREATISVilleurbanne, France; ^3^Information Sciences Institute, University of Southern CaliforniaMarina del Rey, CA, USA

**Keywords:** reproducibility, operating systems, Freesurfer, CIVET, FSL

## Abstract

Neuroimaging pipelines are known to generate different results depending on the computing platform where they are compiled and executed. We quantify these differences for brain tissue classification, fMRI analysis, and cortical thickness (CT) extraction, using three of the main neuroimaging packages (FSL, Freesurfer and CIVET) and different versions of GNU/Linux. We also identify some causes of these differences using library and system call interception. We find that these packages use mathematical functions based on single-precision floating-point arithmetic whose implementations in operating systems continue to evolve. While these differences have little or no impact on simple analysis pipelines such as brain extraction and cortical tissue classification, their accumulation creates important differences in longer pipelines such as subcortical tissue classification, fMRI analysis, and cortical thickness extraction. With FSL, most Dice coefficients between subcortical classifications obtained on different operating systems remain above 0.9, but values as low as 0.59 are observed. Independent component analyses (ICA) of fMRI data differ between operating systems in one third of the tested subjects, due to differences in motion correction. With Freesurfer and CIVET, in some brain regions we find an effect of build or operating system on cortical thickness. A first step to correct these reproducibility issues would be to use more precise representations of floating-point numbers in the critical sections of the pipelines. The numerical stability of pipelines should also be reviewed.

## 1. Introduction

Neuroimaging pipelines are known to generate different results depending on the computing platform where they are compiled and executed (Krefting et al., 2011; Gronenschild et al., 2012). Such reproducibility issues, also known as computing noise, arise from variations in hardware architectures and software versions. The state-of-the-art solution to deal with these issues is to restrict studies to a single computing platform (hardware and software), which has several drawbacks: (i) results may not be reproducible over time, when the computing platform used to produce them becomes obsolete; (ii) the use of High-Performance Computing (HPC) is limited to homogeneous sets of platforms, while available platforms are increasingly versatile; (iii) in some cases, homogenizing computing platforms is not even feasible, for instance when shared databases are processed in different institutions. Before such reproducibility issues can be resolved, a first step is to properly quantify and explain them in various use-cases, which is the objective of this paper.

**Figure 1:**
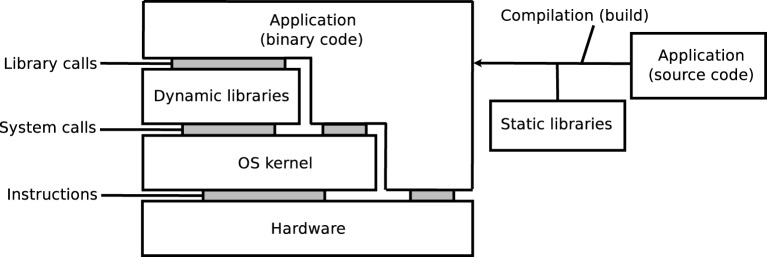
**Source code, compilation, libraries, kernel and hardware**.

As illustrated on Figure 1, the execution of an application depends on its source code, on the compilation process, on software libraries, on an operating system (OS) kernel, and on a hardware processor. Libraries may be embedded in the application, i.e., statically linked, or loaded from the OS, i.e., dynamically linked. The reproducibility of results may be influenced by any variation in these elements, in particular: versions of the source code, compilation options, versions of the dynamic and static libraries (in particular when these libraries implement mathematical functions), or architecture of hardware systems. Some programming languages, for instance MATLAB, Java, Python, Perl, and other scripting languages, additionally rely on a specific runtime software, which can further influence the results.

On GNU/Linux, a dominant OS in neuroimaging (Hanke and Halchenko, 2011) and in HPC^1^, applications rely on the GNU C library, glibc^2^, which includes a mathematical library, libmath. New versions of glibc are released regularly, and subsequently adopted by distributions of the GNU/Linux OS, sometimes several years later. We focus on the differences generated by different library versions, which we call *inter-OS* differences for dynamically-linked applications, and *inter-build* differences for statically-linked applications. *Inter-run* differences, that is, differences between runs of the same application on the same platform may also occur, for instance when applications use pseudo-random numbers (this particular case can be addressed by forcing the seed number used to initialize the pseudo-random number generator).

This paper reports on our experiments with three of the main neuroimaging tools: the FMRIB Software Library (FSL, Jenkinson et al., 2012), Freesurfer (Fischl, 2012), and CIVET (Ad-Dabbagh et al., 2006). We quantify the reproducibility of tissue classification (cortical and subcortical), resting-state fMRI analysis, and cortical thickness extraction, using different builds of the tools, deployed on different versions of GNU/Linux. We also identify some causes of these differences, using library-call and system-call interception. The paper closes with a discussion suggesting directions to address the identified reproducibility issues.

## 2. Materials and methods

### 2.1. Operating systems and applications

Table 1 summarizes the platforms used in our experiments. We used two HPC clusters with Red-Hat-like Linux distributions: (**A**) CentOS release 5.10, running glibc 2.5 released in 2006, and (**B**) Fedora release 20, running glibc 2.18 released in 2013. We installed Freesurfer 5.3.0 and FSL 5.0.6 on these clusters using the 64-bit binaries released on their respective websites^3^^,^^4^. We used the Freesurfer CentOS 4 (**1**) and CentOS 6 (**2**) builds^5^, and the FSL CentOS 5 (**1**) and CentOS 6 (**2**) builds^6^. We compiled and installed CIVET version 1.1.12-UCSF on cluster **A**, and used the same build on cluster **B**.

**Table 1:** **Operating systems and analysis software**.

	**Cluster A**	**Cluster B**
Applications	Freesurfer 5.3.0, build **1**	Freesurfer 5.3.0, build **1** and **2**
	FSL 5.0.6, build **1**	FSL 5.0.6, build **1** and **2**
	CIVET 1.1.12-UCSF, build **1**	CIVET 1.1.12-UCSF, build **1**
Interpreters	Python 2.4.3, bash 3.2.25, Perl 5.8.8, tcsh 6.14.00	Python 2.7.5, bash 4.2.47, Perl 5.18.2, tcsh 6.18.01
glibc version	2.5	2.18
OS	CentOS 5.10	Fedora 20
Hardware	x86_64 CPUs (Intel Xeon)	x86_64 CPUs (Intel Xeon)

Freesurfer releases mainly consist of statically-linked executables and tcsh scripts. Dynamically-linked executables and Perl scripts are also present, in the mni directory where the minc tools are installed. The main differences between the CentOS 4 and CentOS 6 builds are the version of the gcc compiler potentially used to compile them (gcc 3.x on CentOS 4 vs. gcc 4.y on CentOS 6), and the glibc versions embedded in the executables (glibc 2.3 on CentOS 4 vs. 2.12 on CentOS 6). FSL and CIVET consist of dynamically linked executables which depend on libmath and other libraries. FSL also contains Tcl (provided with the FSL release), bash and Python scripts, while CIVET has Perl and bash scripts.

All data movements and task executions on the clusters were performed with the CBRAIN platform for High-Performance Computing (Sherif et al., 2014).

### 2.2. FSL: tissue classification

We used 1.5T T1-weighted MR images from 150 subjects of the International Consortium for Brain Mapping (ICBM, Mazziotta et al., 2001). First, non-brain tissue was removed from the images with FSL BET (Brain Extraction Tool, Smith, 2002), using the default parameters and no options. Next, for cortical and subcortical tissue classification, we used FSL FAST (FMRIB's Automated Segmentation Tool, Zhang et al., 2001) and FSL FIRST (FMRIB's Linear Image Registration Tool, Patenaude et al., 2011) with the default parameters and no options. The experiment was repeated twice in each execution condition to ensure that no inter-run differences were present. Differences were first identified from file checksums. When checksums did not match, classification results were compared using the Dice similarity index (Dice, 1945) (global measure), and the sum of binarized differences across subjects (local measure).

### 2.3. FSL: resting-state fMRI

We used 37 resting-state fMRI (RSfMRI) data arbitrarily selected from an ADNI-GO^7^ dataset (site 130). All fMRI volumes were collected on a 3T Achieva Philips Medical Systems scanner with a gradient echo EPI (TR/TE = 3000/30 ms; Flip Angle = 80.0°; 64.0 × 64.0 inplane isotropic resolution of 3.3125 mm and slice thickness of 3.313 mm). Each RSfMRI dataset contained 140 volumes. Structural images were obtained using a manufacturer T1W MPRAGE sequence.

RSfMRI analysis was carried out using Probabilistic Independent Component Analysis (ICA, Beckmann and Smith, 2004) as implemented in MELODIC (Multivariate Exploratory Linear Decomposition into Independent Components) Version 3.14. We executed MELODIC with FSL build **1**, with the default parameters and different initializations of the random seed: (**a**) fixed, and (**b**) variable (time-based), which is the default. We also varied the dimension of the space of independent components: (**c**) dimension set to 20, and (**d**) automatic dimension detection using the Laplace approximation to the Bayesian evidence of the model order (Minka, 2000; Beckmann and Smith, 2004), which is the default. For variable random seeds, we re-executed MELODIC twice on each cluster to measure the inter-run variability.

We compared results between clusters **A** and **B** by computing the Dice coefficient between their binarized thresholded components, distinguishing the negative and positive parts of the components. As components may not be ordered consistently between **A** and **B**, each component in **A** was matched to the maximally correlated component in **B** using FSL's fslcc. Because this operation is not symmetric, we included Dice coefficients for both **A**–**B** and **B**–**A**. In case **d**, we also compared the number of dimensions detected on cluster **A** vs. cluster **B**.

Then, we analyzed the inter-OS differences between fMRI pre-processing steps. Using fslmaths and fslstats, we computed the mean absolute difference after motion correction, thresholding, spatial smoothing, intensity normalization, and temporal filtering. For motion correction, we also determined the residual rigid transformation *T*_1_*oT*^−1^_2_ at each timepoint, where *T*_1_ and *T*_2_ are the transformations obtained on the different clusters. We measured the norm of the translation vector and the absolute value of the rotation angle of this residual transformation.

### 2.4. Freesurfer and CIVET: surface segmentation and cortical thickness extraction

Cortical thickness maps were generated with Freesurfer and CIVET from the same ICBM dataset used in Section 2.2. In our Freesurfer analysis, we performed all stages of cortical reconstruction using the recon-all pipeline, with qcache option enabled. In our CIVET analysis, we used the default options with the following additional specifications: an N3 spline distance of 200 mm, 12° of freedom for the linear registration, and the tlink metric with a smoothing kernel size of 20 mm FWHM (full-width at half maximum) for the cortical thickness.

Cortical thickness maps were computed in each subject's native space. For Freesurfer, these thickness maps were then resampled to Freesurfer's default fsaverage surface template as a common space, while cortical thickness maps for CIVET were resampled to CIVET 1.1.12's default MNI152 surface template. Resampled thickness files from both Freesurfer and CIVET were imported to the SurfStat MATLAB toolbox (Worsley et al., 2009) for statistical analyses.

To directly compare the effect of build and OS on cortical thickness, a difference score between processing conditions (cluster **A**–**B** or build **1**–**2**) was calculated with SurfStat for the cortical thickness of every subject at every vertex, and a Generalized Linear Model (GLM) was computed consisting simply of the formula Y = 1.

### 2.5. Library and system call interception

We recorded calls to libmath performed by dynamically-linked applications using ltrace^8^ version 0.7.91, patched to facilitate output formatting, and configured to trace children processes created by fork() and clone(). We first completely re-executed a task on each cluster using ltrace's summary mode to list the mathematical functions called by the application. Next, we configured ltrace to record and print the input and output values used in these function calls. In order to avoid excessively large log files, we limited the analysis to a few hours per task, which covered the first few million calls. We also recorded system calls made by applications using strace^9^.

To compare two ltrace traces, we assumed that two executions producing identical results perform the same calls to mathematical functions, in the same order. Traces can then be compared line by line. We classified differences between trace lines in four types. Type-1 differences correspond to functions called on different arguments that produce identical results. They are likely to occur in non-injective functions such as floor() and ceil(). They have little impact on the execution, but are a sign of other differences. Type-2 differences correspond to functions called on different arguments that produce different results. Type-3 differences correspond to functions called on identical arguments that produce different results. They are a sign of implementation differences in the mathematical functions. Type-3 differences usually trigger cascading type-2 and type-3 differences. Mismatches correspond to trace lines where different functions are called. They are a sign that the control flow of the compared conditions differed, for instance due to different numbers of iterations in loops.

## 3. Results

### 3.1. FSL: brain extraction

FSL BET produced identical results for all subjects on clusters **A** and **B**, as well as for builds **1** and **2**.

### 3.2. FSL: cortical tissue classification

**Figure 2:**
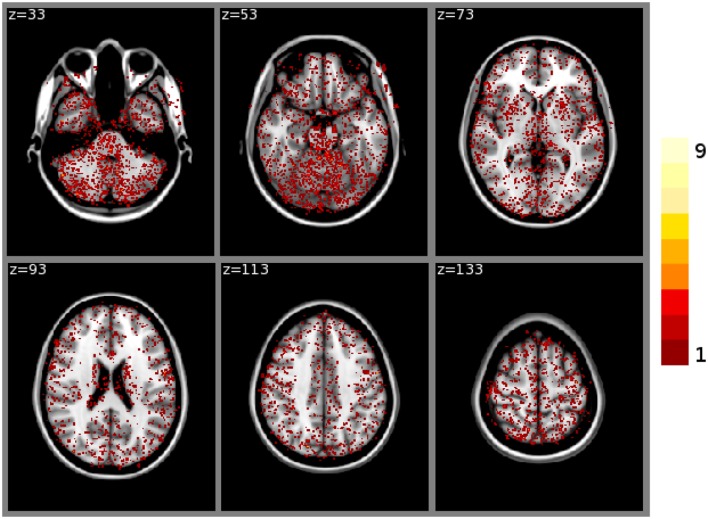
**Sum of binarized differences between cortical tissue classifications obtained on cluster A and cluster B (FSL FAST, build 1, *n* = 150 subjects).** All binarized differences were resampled to the default MNI152 volume template.

FSL FAST cortical tissue classification produced identical results for builds **1** and **2**, but differences between cluster **A** and cluster **B** were found in the classifications of all 150 tested subjects. Table 2 shows the Dice coefficients comparing results obtained on clusters **A** and **B** with FSL FAST, using build **1**. Dice coefficients are very high, indicating very minor differences. Figure 2 shows the sum of binarized differences across segmented subjects. Differences are mostly localized at the interfaces between tissues.

**Table 2:** **Dice coefficients between cortical tissue classifications on cluster A vs. cluster B (FSL FAST, build 1, *n* = 150 subjects)**.

**Tissue**	**Average dice**	**Standard deviation**
Global	0.99973	0.00013
Gray matter	0.99971	0.00014
White matter	0.99973	0.00013
CSF	0.99977	0.00012

**Figure 3:**
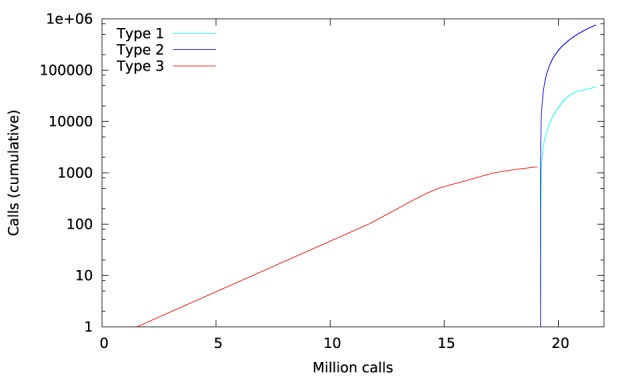
**Cumulative inter-OS differences in FSL FAST**.

Library call interception reveals the cause of these differences. Figure 3 plots a trace of the first 22 million calls to libmath made by FSL FAST to process a randomly-chosen subject of the study. Only log() and expf() were called. The first differences appear at 1.5 million calls: they are type-3 differences in function expf() which manipulates single-precision floating-point representations. Type-1 and type-2 differences appear at 19.2 million calls, both in log() and expf(). No mismatch was found. The following C program excerpt reproduces the first observed type-3 difference:

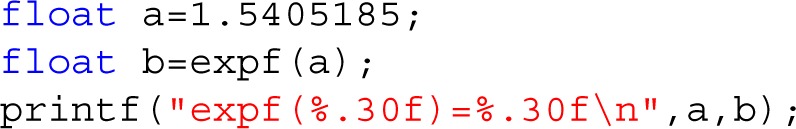



This program prints 30 decimals to display the complete representation of the floating-point numbers. When this representation has less than 30 decimals, printf() pads the displayed string with zeros. With glibc 2.5, this program prints:

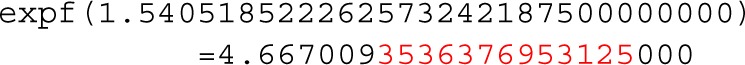



The result produced by expf(), stored in variable b, is encoded as 24 58 95 40 in hexadecimal (little-endian convention). On the other hand, with glibc 2.18, the program prints:

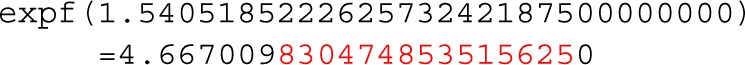



The result produced by expf(), stored in variable b, is encoded as 25 58 95 40 in hexadecimal (little-endian convention): 1 bit is flipped compared to the result obtained with glibc 2.5. These numerical differences, which originate in changing implementation of expf() between glibc 2.5 and 2.18, are a cause of the inter-OS differences in FSL FAST.

### 3.3. FSL: subcortical tissue classification

**Figure 4:**
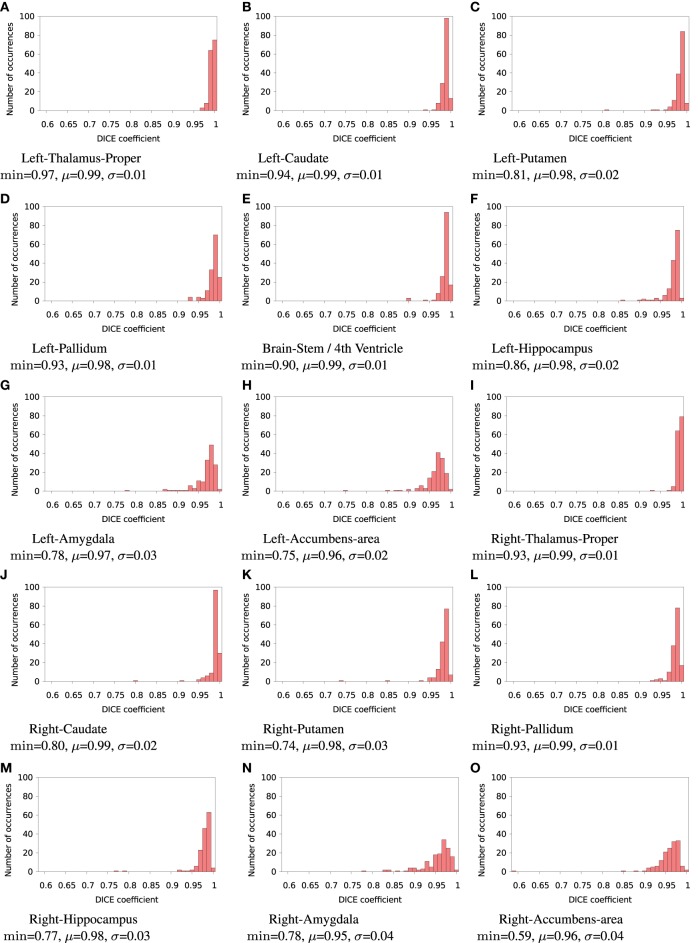
**Histograms of Dice coefficients between classifications obtained on cluster A vs. cluster B with FSL FIRST**. All bins are of size 0.1. min, μ, and σ are the minimum, mean and standard deviation Dice coefficient, respectively.

**Figure 5:**
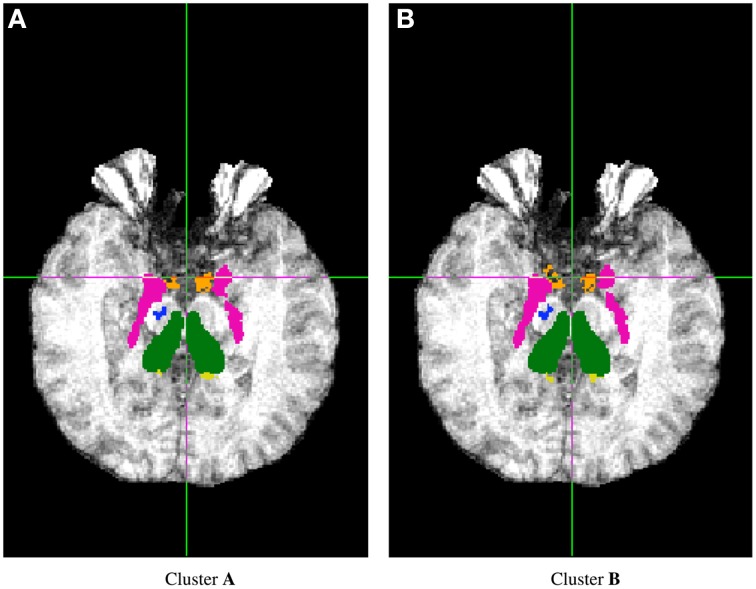
**Sample subcortical classifications with FSL FIRST: subject 260, *Z* = 114**.

FSL FIRST subcortical tissue classification produced identical results for builds **1** and **2**, but differences between cluster **A** and cluster **B** were found in the classifications of all 150 tested subjects. Figure 4 plots the histograms of Dice coefficients for the 15 structures segmented with FSL FIRST, using build **1**. All histograms have a main mode around 0.99, but overall, only 12.7% of the classifications are identical on cluster **A** and cluster **B** (286 classifications out of 2250). Some Dice coefficients are very low, down to 0.59, in particular for small structures such as the amygdalae and the accumbens areas. Figure 5 shows a result sample with Dice coefficients ranging from 0.75 to 0.95.

**Figure 6:**
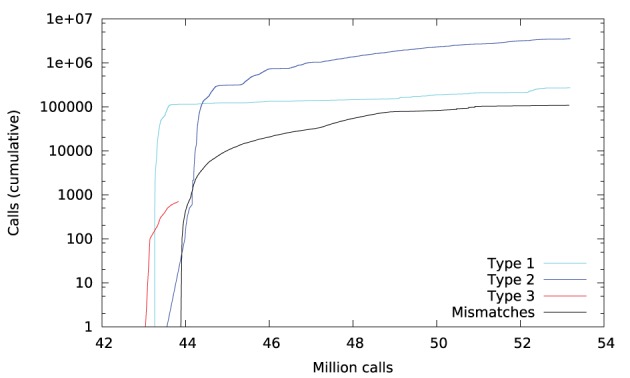
**Cumulative inter-OS differences in FSL FIRST**.

Figure 6 plots a trace of the first 53 million calls to libmath made by FSL FIRST to process a randomly-chosen subject. The trace shows no inter-OS difference until 43 million calls, where type-3 differences start to appear in function cosf(), soon followed by type-1 differences in ceilf() and floorf(), and type-2 differences in cosf(), sinf(), ceilf(), floorf(), and logf(). Mismatches appear at 43.9 million calls, indicating that inter-OS differences have an impact on the control flow of the program. An inspection of the source code shows that the bounds of a few loops are determined from floorf() and ceilf()^10^, which is a plausible explanation for these mismatches.

Type-3 differences come exclusively from function cosf() which manipulates single-precision floating-point representations. The following C program excerpt reproduces the first one:

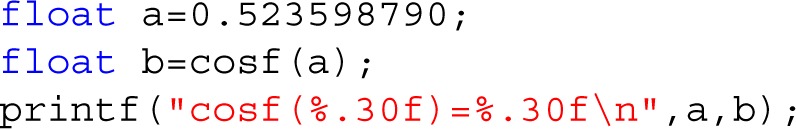



With glibc 2.5, this program prints:

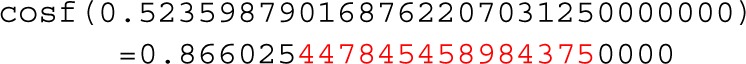



The result produced by cosf(), stored in variable b, is encoded as d8 b3 5d 3f in hexadecimal (little-endian convention). With glibc 2.18, this program prints:

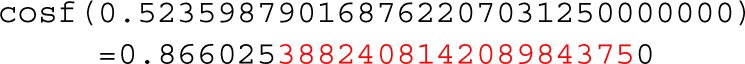



The result produced by cosf(), stored in variable b, is encoded as d7 b3 5d 3f in hexadecimal (little-endian convention): again, 1 bit is flipped compared to the result obtained with glibc 2.5. These numerical differences, which originate in changing implementation of cosf() between glibc 2.5 and 2.18, are a cause of the inter-OS differences in FSL FIRST.

### 3.4. FSL: resting-state fMRI

#### 3.4.1. Variable random seeds

In case **d** (automatic dimension detection), we observed no inter-run differences in the number of detected dimensions, but we found inter-OS differences in 2 subjects out of 37 (47 vs. 48 components and 55 vs. 57 components, respectively).

**Figure 7:**
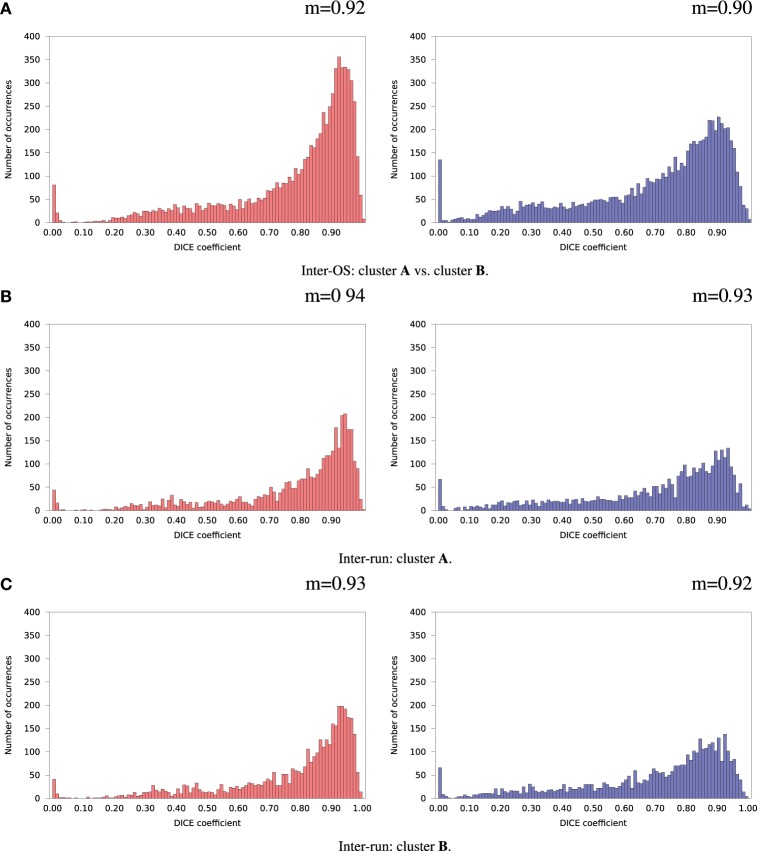
**Histograms of Dice coefficients between matched ICA components.** Variable random seed initialization (case **b**), automatic dimension detection (case **d**). Red histograms, positive components; Blue histograms, negative components; m, mode of the histogram.

**Figure 8:**
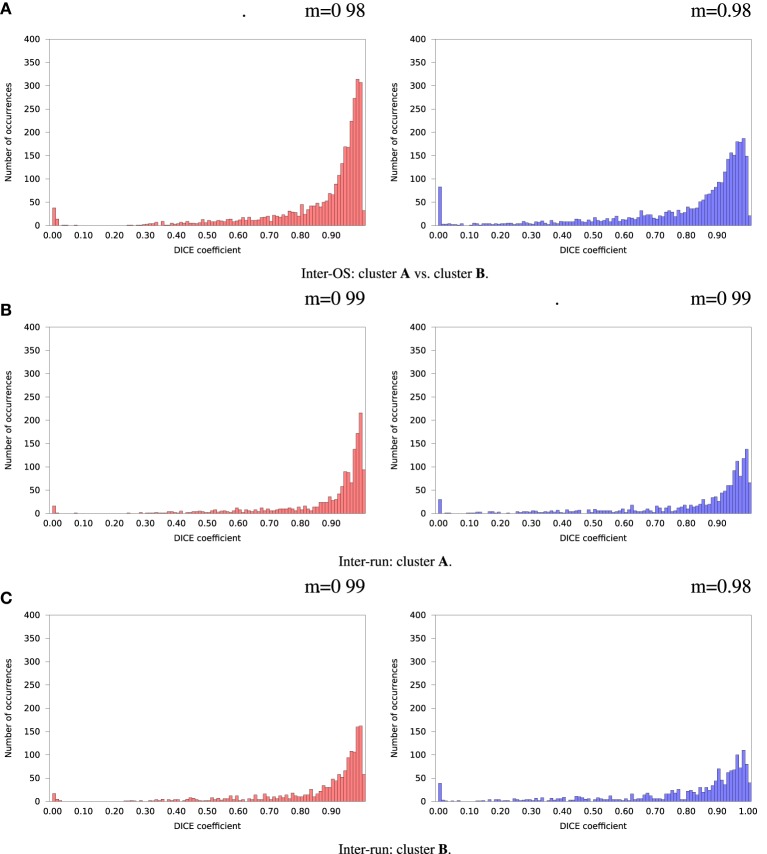
**Histograms of Dice coefficients between matched ICA components.** Variable random seed initialization (case **b**), fixed dimension (case **c**). Red histograms, positive components; Blue histograms, negative components; m, mode of the histogram.

For the remaining 35 subjects, inter-run and inter-OS differences obtained with variable random seeds are shown in Figure 7 for case **d** (automatic dimension detection), and in Figure 8 for case **c** (dimension fixed to 20). All histograms appear bimodal, with a first mode at Dice = 0, and a second around Dice = 0.9. The modes at Dice = 0 correspond to situations where the positive and negative components are inverted, or one of the two compared components has very few voxels. Inter-run and inter-OS differences are significant, and they are of similar magnitude (see modes *m* reported above the graphs).

#### 3.4.2. Fixed random seeds

**Figure 9:**
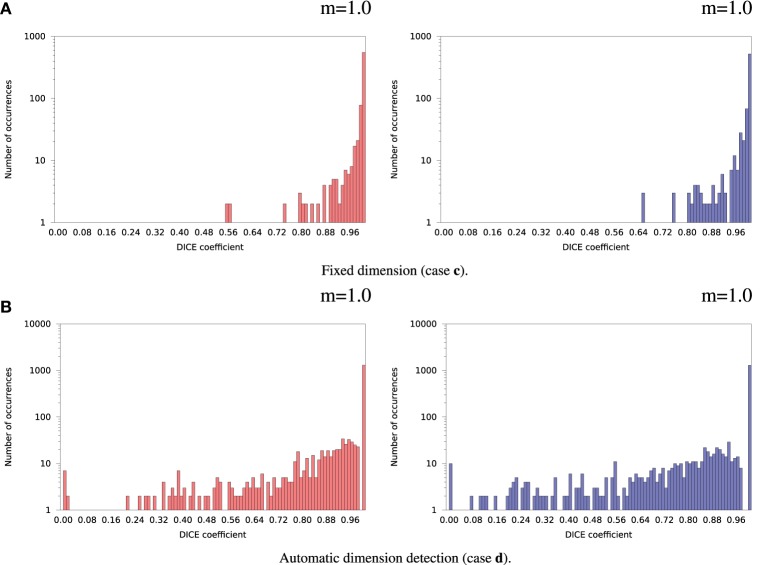
**Histograms of Dice coefficients between matched ICA components on cluster A vs. cluster B (logarithmic scale).** Fixed random seed initialization (case **a**). Red histograms, positive components; Blue histograms, negative components; m, mode of the histogram.

Inter-OS differences in the number of detected dimensions were found in the same 2 subjects as for variable seeds. For the remaining 35 subjects, inter-OS differences obtained with fixed random seeds are shown on Figure 9 for fixed (case **c**) and automatically detected dimensions (case **d**). Inter-OS differences are substantial in both cases, with Dice values lower than 0.9.

**Figure 10:**
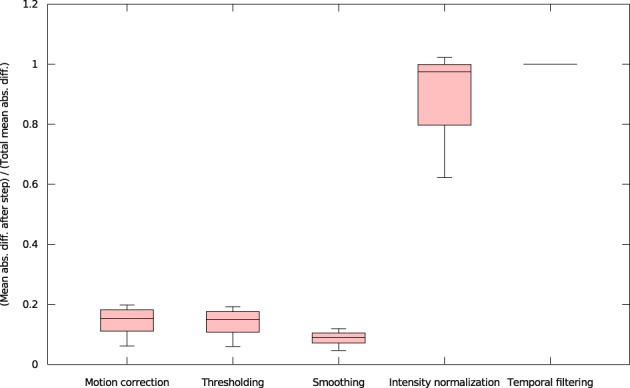
**Mean absolute differences after successive steps of pre-processing, normalized by the mean absolute difference after all pre-processing steps (all 37 subjects)**.

We found that inter-OS differences appear if and only if pre-processed data are different, which occurs in 12 out of 37 subjects. More precisely, inter-OS differences appear if and only if motion-corrected data are different. Figure 10 plots the measured inter-OS mean absolute difference after each main pre-processing step, normalized with the mean absolute difference after all pre-processing steps. We can see that motion correction generates only slight differences, less than 20% of the total difference created by pre-processing. These differences are reduced by spatial smoothing but largely amplified by intensity normalization. Thresholding and temporal filtering have only a minor impact on the global error. Differences in motion correction are quite subtle: residual transformations all have a norm of translation vector below 10^−5^ mm, and rotation angle under 0.096°.

**Figure 11:**
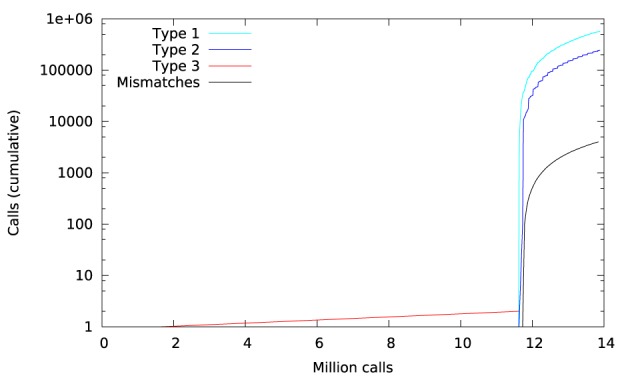
**Cumulative inter-OS differences in FSL mcflirt**.

Figure 11 shows a trace of the first 14 million calls to libmath made by mcflirt to process a randomly-chosen subject. The first inter-OS difference is a type-3, observed at 1.6 million calls in function sinf() which manipulates single-precision floating-point representations. Another type-3 difference in the same function appears at 11.6 million calls, soon followed by type-1 and type-2 differences in ceilf(), cosf(), logf(), sinf(), and floorf(). Mismatches appear at 11.7 million calls, indicating that inter-OS differences have an impact on the control flow of the program. The two observed type-3 differences come from function sinf(). The following C program excerpt reproduces the first one:


float a=0.042260922;
float b=sinf(a);
printf(“sinf(%.30f)=%.30f\n”,a,b);


With glibc 2.5, this program prints:

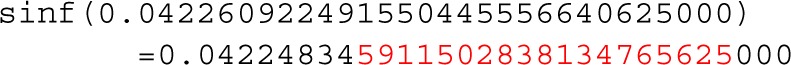



The result produced by sinf(), stored in variable b, is encoded as 9a 0c 2d 3d in hexadecimal (little-endian convention). With glibc 2.18, the program prints:

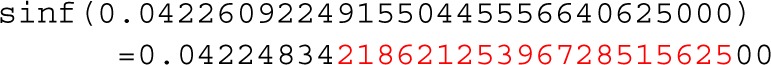



The result produced by sinf(), stored in variable b, is encoded as 99 0c 2d 3d in hexadecimal (little-endian convention): again, 1 bit is flipped compared to the result obtained with glibc 2.5. These numerical differences, which originate in changing implementation of sinf() between glibc 2.5 and 2.18, are a cause of the inter-OS differences in mcflirt.

### 3.5. Freesurfer and CIVET: surface segmentation and cortical thickness extraction

Four subjects were dropped from the results for the following reasons: Freesurfer analysis failed to reach completion (*n* = 3), and missing age information (*n* = 1).

#### 3.5.1. Freesurfer: inter-build differences

**Figure 12:**
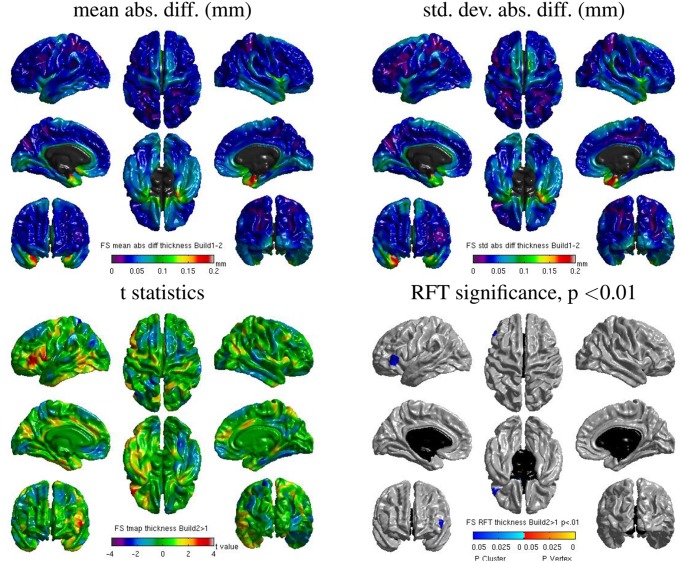
**Surface maps of mean absolute difference, standard-deviation of absolute difference, *t*-statistics and RFT significance values showing regions where the cortical thickness extracted with Freesurfer differs for build 1 and build 2 (both executed on cluster B)**.

Some localized regions of differences were found for Freesurfer build **1** vs. **2** on cluster **B**. Figure 12 shows surface maps of mean absolute difference, standard deviation of absolute difference, *t*-statistics and whole-brain random field theory (RFT) corrections (peaks and clusters) for *n* = 146 subjects at significance value of *p* < 0.01, comparing the cortical thickness values extracted by Freesurfer build **1** and build **2** on cluster **B**. Areas in shades of blue on the RFT map are significant at the cluster (but not peak) level. The cortical thickness values extracted with build **1** are significantly different than with build **2** in the left inferior frontal gyrus at an initial cluster threshold of *p* < 0.01 (family-wise error (FWE) of *p* < 0.05).

#### 3.5.2. Freesurfer: Inter-OS differences

**Figure 13:**
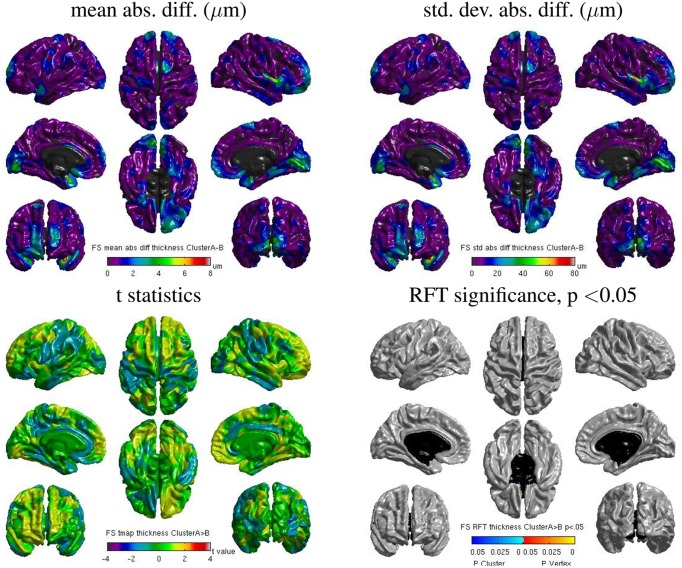
**Surface maps of mean absolute difference, standard-deviation of absolute difference, *t*-statistics and RFT significance values showing regions where the cortical thickness extracted with Freesurfer differs for cluster A and cluster B (both executed with build 1)**.

Despite the static linking of Freesurfer's main executables, we still found small inter-OS differences. Figure 13 shows surface maps of mean absolute difference, standard deviation of absolute difference, *t*-statistics and whole-brain random field theory (RFT) corrections for *n* = 146 subjects at a significance value of *p* < 0.05, comparing the cortical thickness values extracted by Freesurfer build **1** on cluster **A** and cluster **B**. Note the different scales compared to Figure 12. Although no values on the RFT map reach significant levels, the t values do reach upwards of ±2. These residual differences, present in 6 subjects, are introduced by statically-linked executables mri_em_register (2 subjects) and mri_surf2surf (4 subjects). Using strace, we found that these tools open a few libraries from the operating system, including libmath. Differences in these libraries are very likely to create the observed inter-OS differences, although ltrace cannot be used on statically-linked tools to confirm this hypothesis.

#### 3.5.3. CIVET: Inter-OS differences

**Figure 14:**
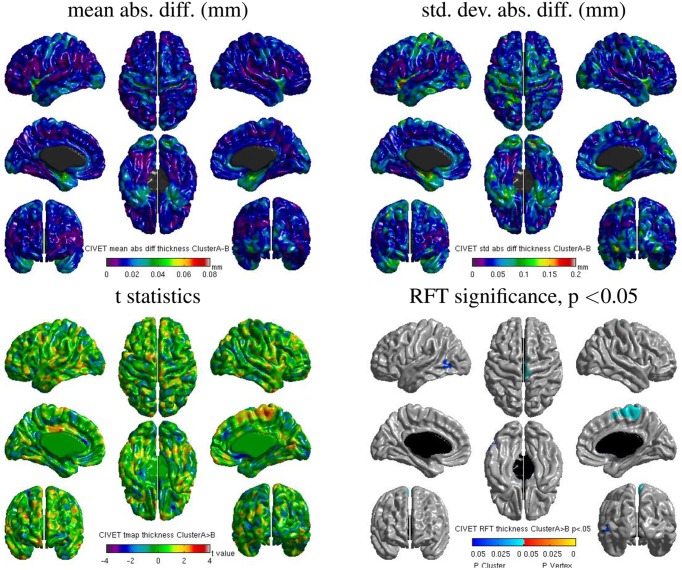
**Surface maps of mean absolute difference, standard-deviation of absolute difference, *t*-statistics and RFT significance values showing regions where the cortical thickness extracted with CIVET differs for cluster A and cluster B (both executed with build 1)**.

We also found some localized regions of differences for CIVET cluster **A** vs. **B**. Figure 14 shows surface maps of mean absolute difference, standard deviation of absolute difference, *t*-statistics and random field theory (RFT) for *n* = 146 subjects at a significance value of *p* < 0.05, comparing the cortical thickness values extracted by CIVET on cluster **A** and **B**. The cortical thickness values extracted on cluster **A** are significantly different than on cluster **B** at an initial cluster threshold of *p* < 0.05 (FWE of *p* < 0.0005 in the right paracentral lobule and FWE of *p* < 0.04 in the left middle temporal region). No significant difference between clusters **A** and **B** was found at a stricter initial cluster threshold of *p* < 0.01.

## 4. Discussion

### 4.1. General conclusions

The implementation of mathematical functions manipulating single-precision floating-point numbers in libmath has evolved during the last years, leading to numerical differences in computational results. While these differences have little or no impact on simple analysis pipelines such as brain extraction and cortical tissue classification, their accumulation creates important differences in longer pipelines such as the subcortical tissue classification, RSfMRI analysis, and cortical thickness extraction.

For cortical tissue classification with FSL, Dice values as low as 0.59 were found between OSes. In RSfMRI, different numbers of components were occasionally found in the two OSes, and the identified components had important differences. Differences in cortical thickness were found for some brain regions as a function of build or OS.

Statically building programs improves reproducibility across OSes, but small differences may still remain when dynamic libraries are loaded by static executables, as observed with Freesurfer. When static builds are not an option, software heterogeneity might be addressed using virtual machines (VMs) as tested in CBRAIN (Glatard et al., 2014), or lighter container environments such as Docker^11^. Specific Linux distributions such as Neurodebian (Halchenko and Hanke, 2012) could be used with these environments to guarantee a wide reproducibility within the community. However, such solutions are only workarounds: differences may still arise between static executables built on different OSes (as seen in our Freesurfer study), or between dynamic executables executed in different VMs.

Although it would not improve numerical stability, a more rigorous way to address reproducibility issues would be to use higher-precision representations of floating-point numbers, and to avoid using functions operating on single-precision numbers (e.g., expf(), cosf(), …). Using double precision would probably address most issues, and the remaining ones could be tackled with quadruple or even arbitrary precision as discussed in Bailey et al. (2012). To limit the resulting performance reduction, precision could be increased only in the code sections creating reproducibility issues.

Identifying such code sections is not trivial though, in particular when pipelines result from a long development process. We showed that library call interception yields accurate information about the functions that are responsible for reproducibility issues in dynamically-linked programs. This technique is, however, extremely heavy in terms of computational overhead and size of the generated traces, and therefore could not be used systematically.

When pipelines produce intermediary result files, a more efficient way to identify suspicious code sections is to compare these intermediary files using some data-specific distance. For instance, using the mean absolute difference between intermediary results produced by FSL pipelines, we were able to quantify the effect of fMRI pre-processing steps on inter-OS reproducibility and to narrow-down the investigation to motion correction. We were also able to identify the tools creating inter-OS differences in Freesurfer.

To conclude, it is clear to us that developers should carefully review the numerical reproducibility and stability of their pipelines using quantitative tests conducted in different execution conditions. However, this could not be done systematically unless a proper platform is available to run such tests and interpret the results. Such a platform could provide benchmarks, virtual execution environments, and analysis tools to help developers identify the cause of observed differences. Frameworks such as testkraut^12^ could be useful in this context.

### 4.2. Limitations

Our results cover some of the main neuroimaging analysis tools (Freesurfer, FSL and CIVET), executed on RedHat-like Linux operating systems which are widely used in neurosciences. To cover a large spectrum of OSes, we used the oldest still-supported version of CentOS and the latest version of Fedora which anticipates on the coming CentOS versions. This encompasses 7 years of glibc development, from version 2.5 in 2006 to 2.18 in 2013, and a much longer range of Linux distributions. For instance, our study gives an idea of reproducibility issues that will arise when upgrading platforms to the recently-released CentOS 7 distribution, which is based on glibc 2.17.

The range of operating systems tested in this study remains, of course, limited. We expect that comparing intermediate glibc versions would only reduce the magnitude of the reported effects. Other Linux distributions, for instance Debian and Ubuntu, are very likely to suffer the same reproducibility issues as long as they are based on glibc. Similar issues are also very likely to occur on non-Linux operating systems, see for instance differences observed between Mac OS 10.5 and 10.6 by Gronenschild et al. (2012).

Our study is limited to compiled application programs. Applications written with interpreted languages such as MATLAB and Python would most likely behave differently. Compilation options were also not considered in this study and are likely to impact the reproducibility of results. For instance, the gcc C compiler has several options that speed-up floating-point operations at the cost of numerical correctness. Using such options to compile programs that are sensitive to small numerical differences is very likely to compromise inter-OS reproducibility, too. Some of the differences observed between Freesurfer builds are likely to originate from the use of different versions of gcc to compile these builds.

### 4.3. Related work

Gronenschild et al. (2012) report the effects of Freesurfer version, workstation type, and OS version on anatomical volume and cortical thickness measurements. Their study was conducted with different versions of Freesurfer (4.3.1, 4.5.0, and 5.0.0). We deliberately chose not to compare different versions of the tested pipelines. Instead, we focused on differences that originate in the system libraries. The Freesurfer versions used by Gronenschild et al. (2012) were dynamically linked (version 5.0.0 was linked statically on Linux, but dynamically on Mac), while the current one (5.3) is statically linked. Thus, the difference reported by Gronenschild et al. (2012) between Mac OS 10.5 and Mac OS 10.6, and between HP and Mac, most likely comes from the use of different system libraries in these platforms. Statically building executables might be seen as a way to address the issues shown by our study shows that it is only a workaround since different builds unsurprisingly yield different results. We also show that these problems are not specific to Freesurfer, but generalize to FSL and to some extent CIVET; it suggests that several other analysis packages are likely to be impacted. Besides, our choice of operating systems (CentOS 5.10 and Fedora 20) encompasses 7 years of glibc development; this gives an idea of how results may evolve in the coming upgrades of HPC clusters to CentOS 7. Finally, we provide an explanation of the causes for inter-OS reproducibility issues; this suggests that these issues may be addressed by using more precise representations of floating-point numbers in some sections of the pipelines.

Krefting et al. (2011) studied the reproducibility of Freesurfer 5.0.0 on Mac OS 10.6, CentOS 4, and SUSE Linux 10.1. They report that the CentOS 5 and CentOS 4 Freesurfer builds gave identical results, but that results obtained with the same build were different across operating systems. This seems in contradiction with our results (we found that different Freesurfer builds give different results). A possible explanation for these differences is that the authors used a dynamically-linked version of Freesurfer 5.0.0, as suggested when they report that different implementations of dynamically linked libraries may explain their findings.

### Conflict of interest statement

The authors declare that the research was conducted in the absence of any commercial or financial relationships that could be construed as a potential conflict of interest.

## Footnotes

^1^http://www.top500.org

^2^http://www.gnu.org/software/libc

^3^http://freesurfer.net/fswiki/Download

^4^http://fsl.fmrib.ox.ac.uk/fsldownloads/fsldownloadmain.html

^5^md5sum: 084d341cdf98305127aaeee48a6f4e0b and 6642289df823ebc27de52af57e9b3989.

^6^md5sum: 4d3a170d2311fa1c7e3cf6efd13f51a5 and 6cf9e3e58b35948416f833a21f495bd8.

^7^http://www.adni-info.org

^8^http://ltrace.org

^9^http://strace.sourceforge.net

^10^See for instance the for loops in method intensity_hist in first.cc, called from do_work

^11^http://www.docker.com

^12^https://testkraut.readthedocs.org/en/latest/index.html
